# Benefit of warm water immersion on biventricular function in patients with chronic heart failure

**DOI:** 10.1186/1476-7120-7-33

**Published:** 2009-07-06

**Authors:** Bente Grüner Sveälv, Åsa Cider, Margareta Scharin Täng, Eva Angwald, Dimitris Kardassis, Bert Andersson

**Affiliations:** 1 Department of Molecular and Clinical Medicine/Cardiology, Wallenberg Laboratory, Institute of Medicine, Sahlgrenska Academy at the University of Gothenburg, Sweden; 2 Institute of Neuroscience and Physiotherapy, Sahlgrenska Academy at the University of Gothenburg, Sweden

## Abstract

**Background:**

Regular physical activity and exercise are well-known cardiovascular protective factors. Many elderly patients with heart failure find it difficult to exercise on land, and hydrotherapy (training in warm water) could be a more appropriate form of exercise for such patients. However, concerns have been raised about its safety.

The aim of this study was to investigate, with echocardiography and Doppler, the acute effect of warm water immersion (WWI) and effect of 8 weeks of hydrotherapy on biventricular function, volumes and systemic vascular resistance. A secondary aim was to observe the effect of hydrotherapy on brain natriuretic peptide (BNP).

**Methods:**

Eighteen patients [age 69 ± 8 years, left ventricular ejection fraction 31 ± 9%, _peak_VO_2 _14.6 ± 4.5 mL/kg/min] were examined with echocardiography on land and in warm water (34°C).

Twelve of these patients completed 8 weeks of control period followed by 8 weeks of hydrotherapy twice weekly.

**Results:**

During acute WWI, cardiac output increased from 3.1 ± 0.8 to 4.2 ± 0.9 L/min, LV tissue velocity time integral from 1.2 ± 0.4 to 1.7 ± 0.5 cm and right ventricular tissue velocity time integral from 1.6 ± 0.6 to 2.5 ± 0.8 cm (land vs WWI, p < 0.0001, respectively). Heart rate decreased from 73 ± 12 to 66 ± 11 bpm (p < 0.0001), mean arterial pressure from 92 ± 14 to 86 ± 16 mmHg (p < 0.01), and systemic vascular resistance from 31 ± 7 to 22 ± 5 resistant units (p < 0.0001).

There was no change in the cardiovascular response or BNP after 8 weeks of hydrotherapy.

**Conclusion:**

Hydrotherapy was well tolerated by all patients. The main observed cardiac effect during acute WWI was a reduction in heart rate, which, together with a decrease in afterload, resulted in increases in systolic and diastolic biventricular function. Although 8 weeks of hydrotherapy did not improve cardiac function, our data support the concept that exercise in warm water is an acceptable regime for patients with heart failure.

## Introduction

Regular physical activity and exercise are well-known cardiovascular protective factors [1]. In patients with heart failure, exercise contributes to enhanced quality of life, improved functional capacity [2] and lower morbidity and mortality [3]. Consequently, it is of importance to offer suitable training methods to achieve optimal individual physical performance. One example is exercise in a thermoneutral warm (34°C) water bath, which could be appropriate for elderly patients with heart failure who find it difficult to exercise on land [4].

However, because warm water immersion (WWI) causes increased venous return and preload [5], it has been questioned whether hydrotherapy is safe for patients with heart failure [6]. Indeed, it has been observed that some patients respond with a decrease in stroke volume during WWI [7]. However, other studies report beneficial acute hemodynamic effects [8,9]. WWI results in peripheral vasodilatation and reduction in systemic vascular resistance [10]. There are also findings of alterations in neurohormonal activity such as reduced sympathetic activity [9].

The primary aim of this study was to investigate, with echocardiography and tissue Doppler, the acute effect of WWI and effect of 8 weeks of hydrotherapy on biventricular function, volumes and systemic vascular resistance. A secondary aim was to observe the effect of hydrotherapy on brain natriuretic peptide (BNP).

We hypothesised that acute WWI should improve biventricular function and that 8 weeks of hydrotherapy should induce a beneficial cardiovascular response.

## Methods

### Patients

Patients were recruited at Sahlgrenska University Hospital, a tertiary referral institution. We enrolled eighteen patients (5 women) with stable chronic heart failure NYHA II-III. The mean age was 69 ± 8 years, left ventricular ejection fraction (LVEF) 31 ± 9%, _peak _VO_2 _14.6 ± 4.5 mL/kg/min and BNP 172 ± 155 ng/L. Exclusion criteria were uncontrolled hypertension, primary valve disease, and pacemaker rhythm. The patients were in a stable condition without changes in medical treatment in the past 2 months. Baseline characteristics are presented in table 1. The protocol consisted of three observed sessions: (1) baseline (acute effect), (2) after 8 weeks without exercise (control period), and (3) after 8 weeks of hydrotherapy, twice weekly, 45 min in a heated pool, 33–34°C at 40–70% of maximal heart rate reserve. Twelve patients completed all three sessions. There were 6 withdrawals after the baseline investigations due to: hip fracture (n = 1), aortic dissection (n = 1), poor compliance (n = 3) and pronounced freezing after WWI (n = 1). The study was approved by the Ethics Committee at the University of Gothenburg, and written informed consent was obtained from all patients.

**Table 1:** Baseline characteristics of the study population

**Characteristics**	All enrolled patients (n = 18)	Hydrotherapy group (n = 12)
Aetiology (ICM/DCM/MV replacement)	13/4/1	11/2/0
Age, years	69 ± 8	69 ± 7
Gender (male/female)	13/5	8/4
NYHA (class I/II/III)	1/8/9	0/7/5
Duration of heart failure (years)	10 ± 7	11 ± 8
Weight (kg)	81 ± 15	83 ± 16
Height (cm)	175 ± 8	175 ± 9
Heart rate (beats/min)	63 ± 12	62 ± 12
Systolic blood pressure (mmHg)	132 ± 21	132 ± 19
Diastolic blood pressure (mmHg)	78 ± 8	79 ± 7
LVEF (%), supine position	40 ± 8	40 ± 7
LVEF (%), standing position	31 ± 9	32 ± 9
BNP (ng/L)	172 ± 155	169 ± 158
Peak oxygen uptake (mL/min/kg)	14.6 ± 4.5	14.0 ± 3.6
β blockers (%)	83	92
ACEI/ARB (%)	94	83
Diuretics (%)	78	75
Statins (%)	67	75
Digitalis (%)	11	8

Data are mean ± SD

ACEI, angiotensin converting enzyme inhibitor; ARB, angiotensin receptor blocker;

DCM, dilated cardiomyopathy; ICM, ischemic cardiomyopathy; LVEF, left ventricular ejection fraction; MV, mitral valve; NYHA, New York Heart Association.

### Echocardiography

The patients were monitored by electrocardiography throughout the observed sessions and examined whilst standing in a slightly tilted position both on land and in a swimming pool for 20–30 min with the water level up to the sternal notch. Transthoracic echocardiography examinations were performed using Siemens Sequoia 512 with a 3v2c transducer (Mountain View, CA, USA), and data were stored digitally on magnetic optical disks. To protect the probe from water, the transducer was placed in a latex stocking. In accordance with the recommendations of the American Society of Echocardiography, LVEF was calculated using the method of discs, modified Simpson rule [11]. Estimation of pulmonary capillary wedge pressure was calculated by a formula using transmitral Doppler and tissue Doppler, as described by Nagueh [12]. Mean arterial pressure was estimated by the following equation: pressure_diastolic_+1/3(pressure_systolic_-pressure_diastolic_). Systemic vascular resistance was calculated as: mean arterial pressure/cardiac output.

Echocardiographic examinations were assessed at the same time of the day on all three occasions and performed and evaluated, in a blind approach, by same investigator (BGS). The intra-patient variability is presented in table 2.

**Table 2:** Intra-patient variability

**Measurements Standing position**	**Intra-patient variability** **A vs B**		
**n = 12**	**CV (%)**	**r**	**p**
SV (mL) land	13.9	0.82	< 0.01
SV (mL) WWI	8.1	0.92	< 0.001
LVEF (%) land	6.9	0.85	< 0.001
LVEF (%) WWI	8.5	0.83	< 0.001
LVEDV (mL) land	13.1	0.88	< 0.001
LVEDV (mL) WWI	12.8	0.84	< 0.01
LV TVTI s land	10.6	0.89	< 0.001
LV TVTI s WWI	7.3	0.83	< 0.01
RV TVTI s land	6.3	0.95	< 0.001
RV TVTI s WWI	10.7	0.77	< 0.01

A: Baseline, B: After 8 weeks of control period without changes in daily lifestyle CV, coefficient of variation; LV, left ventricular; LVEDV, left ventricular enddiastolic volume; r, correlation; RV, right ventricle; SV, stroke volume; TVTI, tissue velocity time integral; WWI; warm water immersion.

### Ventricular long axis function

Ventricular long axis (LAX) function was assessed with M-mode [13] with the beam positioned from the apex to the insertion of the septal and lateral portion of the mitral valve and from the right ventricular (RV) lateral free wall. Pulsed-wave tissue Doppler imaging (TDI) was assessed from the same positions with additional registration from the anterior and inferior walls. Systolic velocities, early diastolic velocities and atrial velocities were obtained [14]. Annular systolic motion was also measured by calculation of the integral (area) of the systolic velocity curves – tissue velocity time integral (TVTI) [15,16].

### Pulsed wave Doppler

Pulsed wave Doppler was carried out for calculation of stroke volume and cardiac output [17] by registration of velocity time integral from left ventricular outflow tract (LVOT) in the five-chamber view. Cardiac output was calculated from the formula: stroke volume = (LVOT area × VTI) × heart rate, where LVOT area was calculated as: 3.14 × (D/2)^2^[18].

With exception of LVEF, measurements were averaged from at least three heart beats.

### Brain natriuretic peptide

For analysis of BNP, blood samples were collected from patients after 30 min in a recumbent resting position. The samples were placed in chilled tubes, centrifuged and frozen at -70°C until plasma concentration was measured using a immunoradiometric method (Shionogi, Osaka, Japan) [19].

### Statistical methods

Data was analysed using SPSS 11 for Windows (Chicago, IL). The differences between the three sessions were analysed with one way ANOVA (analysis of variance). Paired data (land vs WWI) were analysed with the Wilcoxon signed rank test. Relations of paired data were analysed with paired sample T-test. Coefficient of variation (CV) was calculated according to the formula: CV% = s*100/mean, where s (intra-patients error) = SD/√2 and mean = pooled mean value. Data are expressed as mean ± SD. A p-value < 0.05 was considered as statistically significant.

## Results

All patients responded favourably during WWI, and no cardiovascular or other events occurred during the training or investigations session.

### Acute effect of WWI (n = 18)

#### Heart rate and blood pressure

During acute WWI, the heart rate decreased from 73 ± 12 to 66 ± 11 bpm (p < 0.0001) and diastolic blood pressure from 75 ± 11 to 68 ± 12 mmHg (p < 0.01). The mean arterial pressure decreased from 92 ± 14 to 86 ± 16 mmHg (p < 0.01), and systemic vascular resistance from 31 ± 7 to 22 ± 5 resistant units (p < 0.0001).

#### Systolic function

**Figure 1:**
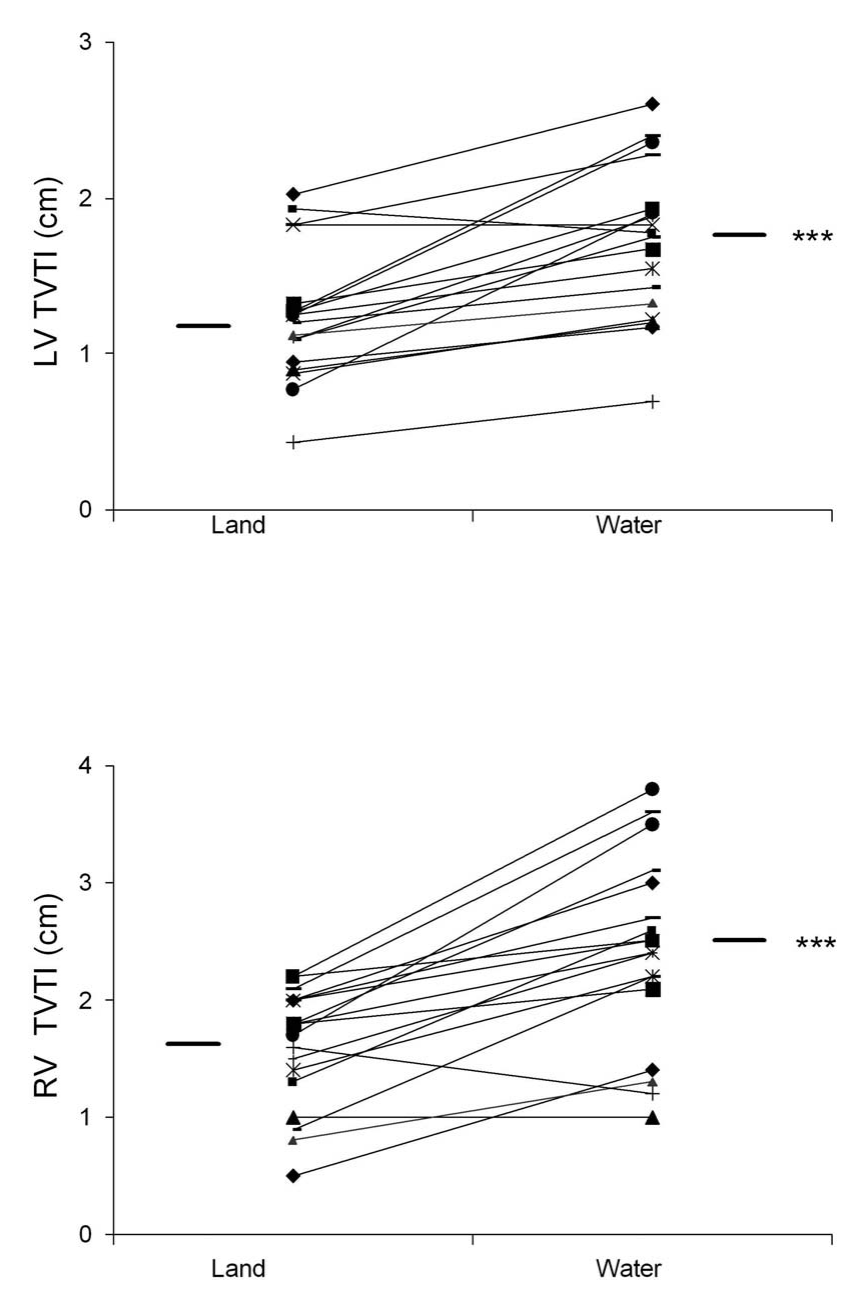
**Improved biventricular function during warm water immersion**. Individual data are shown for each patient on land and in warm water. Mean values are indicated. **1A**. Left ventricular tissue velocity time integral, ***p < 0.001, land vs. warm water immersion. **1B**. Right ventricular tissue velocity time integral, ***p < 0.001, land vs. warm water immersion.

There was a significant hemodynamic response during WWI as reflected in several variables: cardiac output improved from 3.1 ± 0.8 to 4.2 ± 0.9 L/min, and stroke volume from 43.9 ± 13.6 to 64.4 ± 16.5 mL (both p < 0.0001); LVEF improved from 31 ± 9 to 35 ± 8% (p < 0.05); in addition, we found improved LAX function as LV TVTI increased from 1.2 ± 0.4 to 1.7 ± 0.5 cm and RV TVTI from 1.6 ± 0.6 to 2.5 ± 0.8 cm (both p < 0.0001, Figure 1A–B); left atrioventricular plane displacement (AVPD) increased from 5.5 ± 2.1 to 8.2 ± 2.7 mm (p < 0.01).

#### Diastolic function

Early diastolic filling velocity increased from 8.3 ± 1.8 to 9.5 ± 1.8 cm/s (p < 0.05) and E/A ratio from 0.72 ± 0.37 to 1.21 ± 0.75 (p < 0.05).

#### Volumes and filling pressure

Left ventricular end diastolic volume increased from 123 ± 36 to153 ± 56 mL, LV end systolic volume from 85 ± 32 to 100 ± 40 mL and estimated pulmonary capillary wedge pressure from 10.0 ± 5.8 to12.4 ± 5.2 mmHg (p < 0.01, respectively).

**Figure 2:**
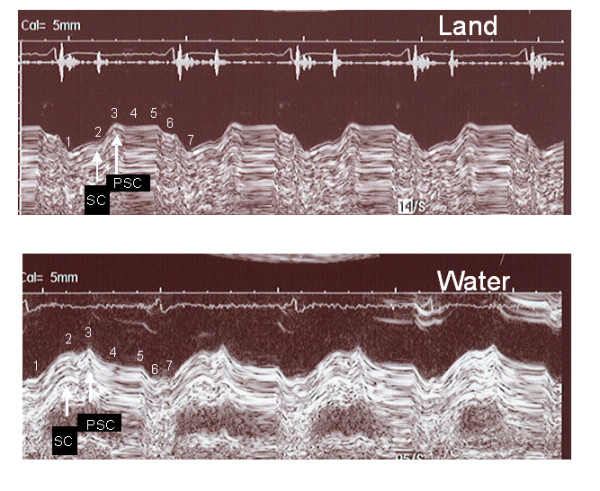
**Decreased postsystolic contraction during warm water immersion**. A patient with left bundle branch block demonstrates a different pattern of the left atrioventricular plane recording on land compared with water. **2A**. Standing position on land, AVPD 3.5 mm, post-systolic contraction 6.1 mm. **2B**. Standing position in warm water, AVPD 8.2 mm, post-systolic contraction 1.4 mm. 1) Start of LV contraction; 2) end of LV contraction; 3) start of early diastolic filling; 4) end of diastolic filling, beginning of diastasis; 5) end of diastasis, beginning of atrial contraction; 6) end of atrial contraction. 7) start of next contraction. AVPD: atrioventricular plane, SC: systolic contraction, PSC: post-systolic contraction.

**Figure 3:**
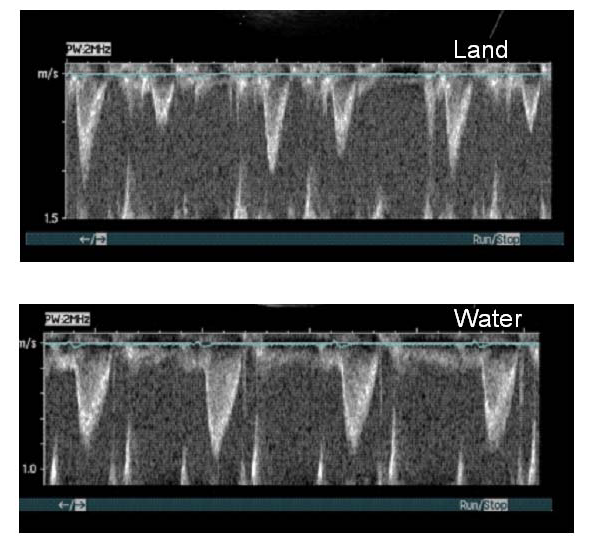
**Irregular heart rate becomes more regular during warm water immersion**. Velocity time integral from the aortic outflow tract is shown for one patient with an irregular heart rhythm on land, which becomes regular during immersion. In addition, stroke volume improved and heart rate decreased. **3A**. On land, LVOT VTI 13.6 cm, stroke volume 57 mL, heart rate 74 bpm, cardiac output 4.2 L/min. **3B**. During warm water immersion, LVOT VTI 20.9 cm, stroke volume 88 mL, heart rate 58 bpm, cardiac output 5.1 L/min. LVOT: left ventricular outflow tract, VTI: velocity time integral.

In three patients with left bundle branch block, we observed pronounced post-systolic contraction when the patients were investigated on land. During WWI, this abnormal contraction was abolished (Figure 2A–B). Furthermore, a more regular heart rate was observed (Figure 3A–B).

### Eight weeks without and with hydrotherapy (n = 12)

**Figure 4:**
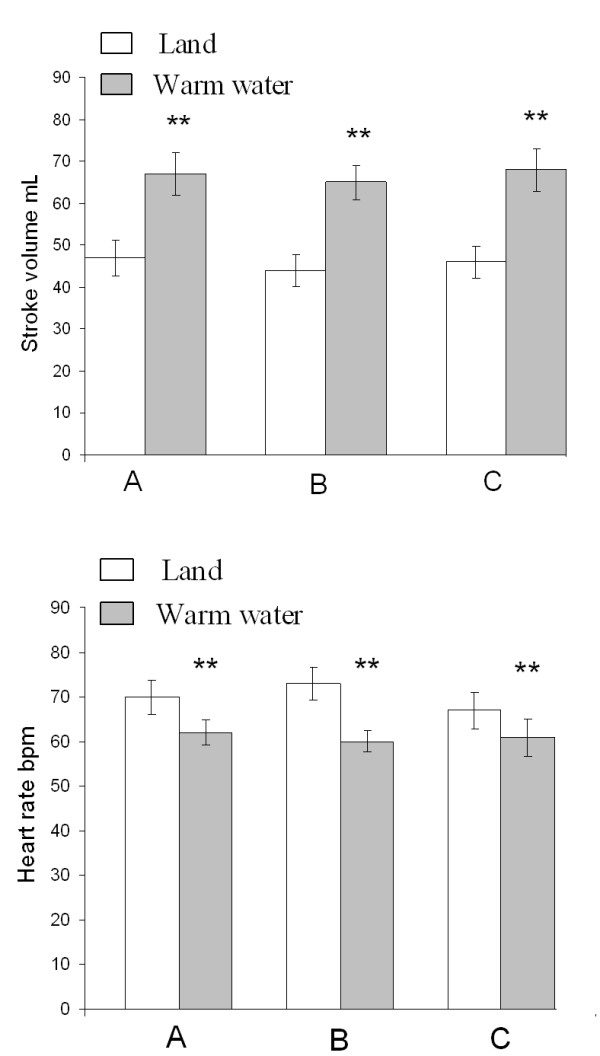
**Echocardiographic data between three sessions on land and during warm water immersion**. **4A**. Effect of warm water immersion with respect to stroke volume. **4B**. Effect of warm water immersion with respect to heart rate. A: Baseline; B: After 8 weeks of control period without changes in daily lifestyle; C: After 8 weeks of hydrotherapy twice weekly. **p < 0.01, mean ± SEM (land vs. warm water immersion).

The effect of WWI was similar during all three sessions with no significant differences after 8 weeks of hydrotherapy (Table 3 and Figure 4). There were no significant differences in BNP during the study period. The levels at baseline, after the control period and after 8 weeks of hydrotherapy were 169 ± 158, 134 ± 102 and 147 ± 125 ng/L, respectively. In addition, exercise and cardiopulmonary function were unchanged (data not shown).

**Table 3:** Echocardiograhic data between three sessions on land and during warm water immersion (n = 12)

	**A**	**B**	**C**
LV TVTI s (cm) land	1.3. ± 0.4	1.3 ± 0.4	1.3 ± 0.4
LV TVTI s (cm) WWI	2.0 ± 0.3**	2.0 ± 0.3**	1.9 ± 0.4**

LVEDV (mL) land	122 ± 40	114 ± 40	110 ± 41
LVEDV (mL) WWI	151 ± 59*	145 ± 56*	142 ± 41**

RV TVTI s (cm) land	1.8. ± 0.5	1.8 ± 0.5	1.9 ± 0.5
RV TVTI s (cm) WWI	2.8 ± 0.6**	2.9 ± 0.7**	2.8 ± 0.8**

PCWP (mmHg) land	9.5 ± 3.6	9.7 ± 4.0	10.1 ± 4.2
PCWP (mmHg) WWI	12.3 ± 6.0**	11.7 ± 4.7*	12.2 ± 4.4*

SVR (RU) land	30 ± 7	30 ± 5	30 ± 7
SVR (RU) WWI	21 ± 5**	23 ± 5**	21 ± 5**

Data are mean ± SD

*p < 0.05; **p < 0.01, land vs. warm water immersion.

There were no significant differences between the sessions.

A: Baseline, B: After 8 weeks of control period without changes in daily lifestyle,

C: After 8 weeks of hydrotherapy twice weekly.

Bpm, beat per minute; LV, left ventricular; LVEDV, left ventricular enddiastolic volume; PCWP, pulmonary capillary wedge pressure; RU, resistant unit; RV, right ventricle; SV, stroke volume; SVR, systemic vascular resistance; TVTI, tissue velocity time integral; WWI; warm water immersion.

## Discussion

This is the first report to show an acute improvement in biventricular function during WWI in patients with stable CHF. In addition, the effect of hydrotherapy on diastolic and systolic cardiac function and volumes has not been studied previously.

The profound decrease in heart rate during immersion was maintained and the hemodynamic response persisted in all three sessions, which is likely to be of importance for tolerance of water immersion.

### Heart rate and ventricular load

The increase in LV volume was caused by hydrostatic pressure and, according to the Bainbridge reflex, would be expected to result in an increase in heart rate [20]. However, we observed a reduced heart rate. Considering the short time of WWI (20–30 min), this was most likely caused by activation of the autonomic nervous system through an enhanced parasympathetic activity [10]. Intravascular volume is controlled by complex systems that involve renal, autonomic, and neurohormonal activities [21-23]. The estimated decrease in afterload was accompanied by a decrease in MAP which is most likely caused by thermally mediated dilatation of skin vessels. A decrease in afterload and unloading of LV ejection is a beneficial aspect of WWI.

### Long axis and diastolic function

We showed that WWI improved several echocardiographic indices of systolic and diastolic function. A lower heart rate alleviates ventricular filling and prolonged diastasis eases myocardial perfusion and is associated with improved systolic function [24]. In our study, effects on heart rate and diastolic function are important to explain why an increase in preload did not cause adverse cardiac effects in heart failure patients.

For many years, the M-mode technique was the established method to study LAX function. However, with the development of newer and relatively preload-independent techniques such as TDI and strain rate, the interest in measuring LAX function has increased dramatically. We have recently shown that LAX function is of major importance for long-term survival [25]. In this present study, we investigated LAX function using: 1) M-mode, which measures amplitudes of atrioventricular plane motion, and 2) TDI, which measures myocardial velocities. Although the M-mode technique showed a significant increase in systolic amplitude, the systolic velocity was unaffected when measured by TDI. This could be explained as TDI is less dependent on preload [26,27]. Nevertheless, both RV and LV TVTI increased, which demonstrate enhanced LAX function. When TVTI is applied, amplitudes of myocardial movements are recorded, giving similar information as obtained with M-mode echocardiography. M-mode and TVTI are therefore closely connected. Compared with myocardial velocities, we found that AVPD with M-mode and TVTI increased significantly during WWI.

An abnormal post-systolic contraction in patients who suffered from left bundle branch block was shown to be normalized by WWI. Post-systolic contraction is a delayed ejection motion of the myocardium [28], a phenomenon related to ischemia [29] and intra-ventricular dyssynchrony [30].

### Echocardiographic protocol

Our experimental protocol was comfortable for the patients compared with other studies where patients have been exposed to heart catheterization [31] or investigated with transesophagal echocardiography [6]. Echocardiography is not normally performed in a standing position, but this allowed us to study cardiac effects in a position that is commonly adopted during hydrotherapy.

### Hydrotherapy

Exercise is an important complement to medical treatment, to prevent and reduce symptoms of heart disease, however, regular exercise is required to maintain a positive result [1]. Controlled trials have shown favourable effects after exercise in CHF, leading to improved physiological and psychological effects [2]. Nevertheless, in this present study we did not observe any improvement in cardiac function, exercise tolerance or peak VO_2 _after 8 weeks of hydrotherapy. One explanation for this could be insufficient exercise intensity (40–70% of maximal heart rate reserve). It has been shown that hydrotherapy three times weekly improves functional capacity compared with a deterioration in patients randomized to a control group [4]. Unpredictably, patients in our study maintained their physical capacity during the control phase. One reason could be that they unconsciously became more active ahead of the training regime because of the extra attention they received.

### Brain natriuretic peptide

Plasma concentrations of natriuretic peptides have been shown to be powerful predictors of mortality and disease severity in patients with CHF [32]. Long-term treatment with angiotensin-converting enzyme inhibitors and beta-blockers have been reported to reduce plasma levels of BNP [33,34]. Despite optimal medical treatment, the patients in our study had raised BNP levels. In agreement with the measured unaltered cardiac function BNP remained unchanged after the hydrotherapy

## Limitations

This study has some limitations. We chose a follow-up period before the exercise intervention. As in other training studies, it is impossible to use proper blinded controls. The study population is small. We did not record actual intracardiac pressures, vascular resistance or neurohormone levels. Nevertheless, systolic and diastolic responses of acute WWI were identical in all three sessions, showing that the positive hemodynamic effects were reproducible.

## Conclusion

Under the conditions used in this study, hydrotherapy was well tolerated. The main observed cardiac effect during acute WWI was a reduction in heart rate, which, together with a decrease in afterload, resulted in increases in systolic and diastolic biventricular function, despite an increased preload. Although 8 weeks of hydrotherapy did not improve cardiac function, the exercise form is a safe method to be used during physical training and rehabilitation of stable heart failure patients.

## Abbreviations

AVP: atrioventricular plane; BNP: brain natriuretic peptide; EF: ejection fraction; LAX: long axis; LV: left ventricular; LVOT: left ventricular outflow tract; RV: right ventricular; TDI: tissue Doppler imaging; VTI: velocity time integral; TVTI: tissue velocity time integral; WWI: warm water immersion.

## Competing interests

The authors declare that they have no competing interests.

## Authors' contributions

Each author has contributed significantly to this submitted work. BGS contributed to the design of the study, participated in the cardiopulmonary exercise test, performed the ultrasound investigations, measurements, calculation and statistical analysis, interpretation of data, wrote the manuscript and was responsible for the final version. ÅC contributed to the design of the study, trained the patients in warm water, and contribute to important intellectual content. MST participated in the ultrasound performance, and contributed to important intellectual content. EA participated in the cardiopulmonary exercise test and collected the blood sample. DK was responsible for the safety during the cardiopulmonary exercise test. BA contributed to the design of the study, interpretation of data, revised the article for important intellectual content. All authors read and approved the final manuscript.
